# Prevalence of Masturbation and Associated Factors Among Older Adults in Four European Countries

**DOI:** 10.1007/s10508-021-02071-z

**Published:** 2021-11-09

**Authors:** Nantje Fischer, Cynthia A. Graham, Bente Træen, Gert Martin Hald

**Affiliations:** 1 Department of Psychology, University of Oslo, Gaustadalleén 30 A, 0373 Oslo, Norway; 2 Department of Psychology, Faculty of Environmental and Life Sciences, University of Southampton, Southampton, UK; 3 Department of Public Health, University of Copenhagen, Copenhagen, Denmark

**Keywords:** Masturbation, Solitary sexual activity, Older adults, Sexual attitudes, Sexual satisfaction, Cross-cultural

## Abstract

Solitary sexual activity is a free, safe, and accessible way to experience sexual pleasure. Despite these advantages, research on masturbation in later life is highly understudied. Using data from a cross-sectional probability-based survey of 3816 European adults (mean age 67 years; range 60–75 years), we explored several sociodemographic, health, attitudinal, and sexual behavioral factors associated with reported masturbation frequency. Across all countries, between 41% and 65% of men and 27% and 40% of women reported any masturbation in the preceding month. Satisfaction with sexual activity and attitudes related to disapproval of sex without love were significant predictors of reported masturbation in almost all countries and in both genders. Age, education, self-perceived health, and depression were for the most part predictive of men’s reported masturbation, but not women’s. Generally, those believing sex is beneficial to older people were more likely to masturbate, while less permissive attitudes decreased the likelihood of reporting masturbation. To improve healthy sexual aging, misinformation about masturbation and sexual attitudes in older people need to be addressed.

## Introduction

Although there has been an increasing interest in older adults’ sexual behavior, most research to date has focused on partnered sexual activity and not on solitary sexual behavior (Bell et al., 2017; DeLamater, 2012). A recent review on sexual activity after aged 60 found that of all included studies, 74% investigated sexual intercourse activity, while only 30% assessed masturbation (Bell et al., 2017). In later life, it becomes increasingly common that one partner dies or that a partner’s decreased health and sexual function constrain an individual’s sexual activity, satisfaction, and desire (Fischer et al., 2018; Iveniuk & Waite, 2018; Kontula & Haavio-Mannila, 2009; Rosen et al., 2016; Stroope et al., 2015). Solitary sexual activity has the advantage of providing sexual pleasure independent of a partner’s availability, health, and sexual function (Dekker & Schmidt, 2003; Hinchliff et al., 2018; Kontula & Haavio-Mannila, 2003). Further, among both younger and older women masturbation seems to be related to more consistent orgasms compared with partnered sex (Dekker & Schmidt, 2003; Howard et al., 2006). Despite these advantages, there is surprisingly little research assessing masturbation activity among older populations. The few studies addressing solitary sexual activity in later life have focused solely on a small number of sociodemographic variables (e.g., age, sex, relationship status) and some specific health factors (e.g., hypertension, arthritis, diabetes) (Corona et al., 2010; Lee et al., 2016; Lindau et al., 2007; Palacios-Ceña et al., 2012; Papaharitou et al, 2008; Schick et al., 2010). Consequently, research on the relationships between sexual attitudes, sexual behavioral factors, and masturbation among aging adults is particularly lacking.

### Sociodemographic and Health Factors Associated with Masturbation

A common finding in sexuality and aging research is that the prevalence of reported masturbation decreases as people age (Lee et al., 2016; Lindau et al., 2007; Mercer et al., 2013; Palacios-Ceña et al., 2012; Schick et al., 2010). In the European Male Ageing Study, a population-based survey of 3369 men (40–79 years) from eight European countries, masturbation reported at least once in the past month decreased from 36% in the age cohort 60–69 years to 25% among men aged 70 and above (Corona et al., 2010). Apart from age, research has revealed important gender differences, with women generally reporting less masturbation than men (Kontula & Haavio-Mannila, 2003; Lee et al., 2016; Lindau et al., 2007; Oliver & Hyde, 1993; Palacios-Ceña et al., 2012; Papaharitou et al., 2008; Richters et al., 2014). For example, in the most recent wave of the population-based British National Survey of Sexual Attitudes and Lifestyles, more than twice as many men (53%) in the age group 55–64 years reported masturbating in the past month than women (19%) (Mercer et al., 2013). The respective proportions among men and women aged 65–75 years were 33% and 10%, respectively.

Higher levels of education may be associated with masturbation through better access to information, health literacy, and sex education, which possibly diminish negative assumptions and fears related to masturbation (Gerressu et al., 2008; Kontula & Haavio-Mannila, 2003; Richters et al., 2014). For instance, Kontula and Haavio-Mannila (2003) found that Finns with higher levels of education felt less guilt about masturbation, perceived masturbation less often as unhealthy, and masturbated more often than those with lower levels of education. In addition to the level of education, it may be important in which decade and cultural context older adults received their schooling. Cross-European differences in sex education and country-specific political and religious forces are likely to also influence masturbation habits (Francoeur & Noona, 2004; Kontula & Haavio-Mannila, 2003).

The few studies assessing the relationship between health factors and masturbation have yielded inconclusive and sometimes contradictory findings (Das, 2007; Gerressu et al., 2008; Lee et al., 2016; Lindau et al., 2007; Schick et al., 2010). In a US nationally representative sample of men aged 50 to 107 years, men with poorer health were more likely to report masturbation compared to men with good self-perceived health (Schick et al., 2010)*.* Other studies, however, have reported no significant association between self-reported health and masturbation and only inconsistent findings between masturbation and specific health conditions (e.g., diabetes, high blood pressure; see Lee et al., 2016; Lindau et al., 2007). Studies among older women have suggested either a lower likelihood of masturbation among those with poorer health (Lindau et al., 2007), or nonsignificant associations between self-reported health and masturbation (Lee et al., 2016; Schick et al., 2010).

### Sexual Behavior and Satisfaction Associated with Masturbation

A commonly discussed question in the literature is whether masturbation is a substitute for, or a supplement to, partnered sexual activity (Gerressu et al., 2008; Regnerus et al., 2017). The former perspective (termed the “compensatory model”) describes masturbation as a means to release sexual tension if partnered sex is not satisfying, if one partner desires more frequent sex, or if an individual lacks access to a partner (Das et al., 2009; Regnerus et al., 2017). Indeed, among both younger and older adults, not being in a current relationship has been linked to higher masturbation frequency (DeLamater & Moorman, 2007; Regnerus et al., 2017; Rowland et al., 2020; Schick et al., 2010). In contrast, the second perspective (termed the “complementary model”) suggests that masturbatory activity accompanies or reinforces partnered sexual activity and assumes that partnered sexual activity will lead to enhanced solitary sexual behavior (Regnerus et al., 2017). Although both perspectives have received some support (Carvalheira & Leal, 2013; Das, 2007; Das et al., 2009; Dekker & Schmidt, 2003; Gerressu et al., 2008; Kontula & Haavio-Mannila, 2003), evidence of the association between partnered sexual activity and masturbation frequency is not unequivocal (Regnerus et al., 2017; Rowland et al., 2020). For example, using data from the second wave of the UK Natsal survey of adults (aged 16 to 44), Gerressu et al. (2008) found a gender-specific pattern where, among men, more frequent intercourse decreased the likelihood of reporting masturbation (in keeping with the compensatory model), while the opposite relationship was found among women (consistent with the complementary model). Other studies have found only weak associations between partnered and solitary sexual activity (Långström & Hanson, 2006; Pinkerton et al., 2003; Rowland et al., 2020). Overall, findings suggest the relationship between solitary and partnered sexual activity is complex and likely strongly influenced by other factors, such as individuals’ sexual socialization (Das et al., 2009) and satisfaction with the frequency of partnered sex (Regnerus et al., 2017).

As masturbation is a self-determined and available way of obtaining physical pleasure (Dekker & Schmidt, 2003), one would expect that frequent sexual self-gratification would result in greater sexual satisfaction. Many studies have found, however, the reverse to be true (Ayalon et al., 2019; Brody & Costa, 2009; DeLamater & Moorman, 2007; Lee et al., 2016; Rowland et al., 2020; Velten & Margraf, 2017). In a representative Swedish study of 2810 adults aged 18–74 years (Brody & Costa, 2009), masturbation frequency was negatively related to sexual and relationship satisfaction among both men and women, independent of penile–vaginal intercourse activity and age. Moreover, masturbation was also linked to lower life satisfaction in men. These seemingly paradoxical associations might be related to the long-standing condemnation and stigmatization of masturbation that still elicits guilt, shame, and feelings of sexual inadequacy (Baćak & Štulhofer, 2011; Coleman, 2003; Hinchliff et al., 2018). Qualitative studies among younger adults indicate a substantial diversity of views on masturbation and provide some insights into the contradictious and conflicting experience of masturbation being both pleasurable and shameful (Hogarth & Ingham, 2009; Kaestle & Allen, 2011). For example, Kaestle and Allen found that while young adults were discovering the physical pleasure of solo sex the exploration was often accompanied by feelings of shame and awkwardness, and a subsequent effort to balance those conflicting feelings.

### A Cross-European Perspective

Although in recent years attitudes toward masturbation have become more liberalized, masturbation still seems stigmatized and tabooed (Baćak & Štulhofer, 2011; Carvalheira & Leal, 2013; Coleman, 2003; Dekker & Schmidt, 2003; Francoeur & Noonan, 2004). This may be especially true among older populations that were socialized in different generations and sociocultural environments. It would be expected that differences in gender roles and deep-rooted traditional sociocultural pressures in regulating sexuality may have created different patterns of autoerotic behavior across Europe (Baćak & Štulhofer, 2011; Francoeur & Noona, 2004; Katz-Wise & Hyde, 2014). Portugal, along with other South European countries (e.g., Italy and Spain), are essentially Latin communities, with similar views on sexuality and gender norms (Francoeur & Noona, 2004). The southern European countries share the social influence of the Catholic Church that condemns autoerotic behavior and maintains prevailing sexual double standards (Bajos & Marquet, 2000; Bozon & Kontula, 1998; Francoeur & Noona, 2004). Even though a recent modernization of Portuguese society has led to changes in views on gender roles and sexuality, the traditional and religious beliefs may still remain an influence on the older population (Francoeur & Noona, 2004). The northern European countries, in contrast, are viewed as being more sexually open-minded and permissive (Francoeur & Noonan, 2004; Træen & Kvalem, 1996; Træen & Lewin, 2008). For example, Denmark was the first country in the world to legalize written pornography in 1967 and pornographic pictures in 1969 (Hald, 2007).

Regarding gender equality, the Nordic countries also seem to stand out (European Institute for Gender Equality, 2017; Francoeur & Noonan, 2004; World Economic Forum, 2017). In 2015, Sweden, Denmark, and Finland were ranked as the top three of 28 EU Member States in achieving gender equality (European Institute for Gender Equality, 2017). These sociocultural variations along a North–South European gradient may thus be reflected in different patterns of masturbation across Europe. Previous research that compared masturbation habits between North and East Europe found more frequent levels of masturbation in the liberal Nordic countries (Finland and Sweden) compared to the more sexually restrictive and repressive former Soviet Union (Kontula & Haavio-Mannila, 2003). A study of 40–79-year-old men from eight European countries (Corona et al., 2010) indicated that men from Northern and Central Europe (Belgium, Sweden, UK) were about twice as likely to report having masturbated at least once in the past month than men from Southern Europe (Italy, Spain) (58–66% vs. 28–30%). More gender egalitarian countries may also be characterized by lower gender gaps in reported masturbation.

To date, large-scale probability-based surveys on men’s and women’s masturbation have been limited (Das et al., 2009; Gerressu et al., 2008; Regnerus et al., 2017) and particularly scarce among older adults (Træen et al., 2019). The purpose of the current study was to identify sociodemographic, health, sexual behavior, and attitudinal factors associated with solitary sexual activity among adults aged 60–75 years in Norway, Denmark, Belgium, and Portugal. Although some of these correlates have been studied in younger populations, it was expected that these previous findings may not be applicable to aging women and men. As older adults have been socialized in more sexually restrictive and repressive environments than current younger generations, they may have more internalized stigmatization, fears and guilt when practicing self-stimulation (Štulhofer et al., 2018), which may influence their masturbation habits. To our knowledge, this is the first multi-national study exploring correlates between masturbation and sociodemographic variables (age, education, relationship status, religiosity), health variables (self-perceived physical health, depression), as well as factors related to older adults’ sexual lives (sexual activities, sexual perceptions, and attitudes toward sexuality).

## Method

### Participants and Procedure

In 2016, a study of sexual function and sexual well-being in older European adults was conducted. For the current article, we analyzed the data from four national probability-based samples of individuals aged 60–75 years in Norway, Denmark, Belgium, and Portugal. Data were collected between October 2016 and February 2017 by Ipsos, a polling agency with locally based firms in each country. The recruitment of participants occurred in two stages. First, participants were randomly contacted by telephone and asked to participate. Every country except Portugal had national and updated telephone registers, allowing random sampling in Norway, Denmark, and Belgium. As there exists no complete national telephone register in Portugal, multistage stratified probability sampling common for public opinion surveys in this country had to be used. More detailed information of the sampling procedure has been provided elsewhere (Træen et al., 2019).

Second, for those who agreed to participate Ipsos registered the personal information (names and addresses) and sent them an anonymous questionnaire for self-completion with a freepost envelope. Prospective participants in Norway, Denmark, and Belgium received postal reminders about a week after they got the questionnaire. The 2000 previously recruited participants in Portugal were reminded by phone. However, only three quarters of these participants (*n* = 1498) could be reached again and among those, another 561 declined participation after they had received the mail survey. The Ipsos provided response rates were calculated by dividing the net sample by those who agreed to participate and received a questionnaire, and this resulted in a response rate of 68% in Norway, 57% in Belgium, 52% in Denmark, and 26% in Portugal.

### Measures

Age was assessed by year of birth and later recoded into three age categories (1 = 60–64, 2 = 65–69, and 3 = 70–75 years). Relationship status was measured by asking: “Do you currently have a steady/ committed relationship with anybody? A steady/ committed relationship also includes married/ cohabiting persons.” Response options were 1 = yes, 2 = no, and 3 = unsure. Twenty-one participants checked the category “unsure” and were subsequently dummy coded into the “no” category (2 + 3 = 0).

Education was assessed by asking: “What is your highest level of formal education?” To reflect the educational system in each country, response options differed somewhat across countries. To allow for comparisons across countries, the response categories were recoded into three matching levels of education: 1 = primary (6–8 years at school), 2 = secondary (9–10 years or 12–13 years at school), and 3 = tertiary (college, lower university level (e.g., Bachelor’s degree) to higher university level (e.g., Master’s degree, Ph.D.).

Religiosity was measured by the following question: “Apart from special occasions such as weddings, funerals and baptisms, how often do you attend services or meetings connected with your religion?” Responses were 1 = never, 2 = less than once a year, 3 = once a year, 4 = twice a year, 5 = once a month, 6 = once every two weeks, and 7 = once a week or more.

Self-estimated health was indexed via the following item: “In general, would you say your health is:” Scores were 1 = excellent, 2 = very good, 3 = good, 4 = fair, and 5 = poor. Depression during the past four weeks was measured with a brief psychometrically validated depression scale (SCL-90-DEP; Søgaard & Bech, 2009). The 6 items (e.g., “Feelings of worthlessness,” “Feeling lonely,” “Blaming yourself for things,” etc.) were rated on a 5-point scale (1 = not at all to 5 = extremely), with higher scores reflecting higher levels of depression. The reliability of the scale in this study was acceptable (Cronbach’s *α*_by country_ = 0.78–0.82).

Satisfaction with sexual activity was assessed with the question: “How satisfied are you with the current level of sexual activity in your life, in a general way?” Responses, which ranged from 1 = very satisfied to 5 = very dissatisfied, were reverse-recoded, so that higher scores reflected higher sexual satisfaction.

Intercourse frequency was assessed by a one-item indicator, used in the English Longitudinal Study of Ageing (ELSA; Lee et al., 2016), “How many times have you had or attempted sexual intercourse (vaginal, anal, or oral sex) during the past month?” Responses were made on a 7-point scale (1 = none to 7 = more than once a day).

Sexual attitudes were assessed using 6 items retrieved from the ELSA Sexual Relationship and Activities Questionnaire (SRA-Q; http://www.elsa-project.ac.uk). Responses were made on a 5-point Likert scale ranging from 1 = strongly agree to 5 = strongly disagree. Attitudes reflecting the idea that sex is legitimized by love were assessed with two items (*r*_men_ = 0.44–0.63; *r*_women_ = 0.39–0.62): “Having one-night stands is wrong,” and “A married person having sexual relations with someone other than their spouse is wrong.” The items were averaged into a composite indicator and reverse-recoded, so higher scores indicated negative feelings about having sex without love. The following two items were used to tap into feelings that contemporary society is too sexualized (*r*_men_ = 0.40–0.50; *r*_women_ = 0.35–0.47): “There’s too much sex in the media nowadays,” and “Young people today start having sex too early.” The two items were averaged and scale scores reverse-coded, so that higher scores reflected less liberal views about sexuality in modern society. Attitudes toward sexuality and aging were indexed by two separate items. One item assessed participants’ evaluation of older adults’ sexual ability, compared with younger ages (“The ability to have sex decreases as a person grows older”). The scale scores were reverse-coded, with higher scores reflecting the belief that sexuality decreases with increasing age. The other item asked participants to evaluate the beneficial aspects of sexual activity in later life (“Being sexually active is physically and psychologically beneficial to older people”). The responses were reversed-recoded, with higher scores indicating more positive attitudes toward sexuality at older age.

Masturbation activity was measured by a one-item indicator, also used in the ELSA (Lee et al., 2016), “How often did you masturbate in the past month?” The response options were 1 = none, 2 = once in the past month, 3 = 2 or 3 times in the past month, 4 = once a week, 5 = 2 or 3 times a week, 6 = once a day, and 7 = more than once a day.

### Statistical Analysis

All statistical analyses were performed using IBM SPSS version 27.0 statistical software package. In order to adjust for recruitment-based distortion in sex, age, and region, census-based post hoc weighting was applied for each country. Various group differences were estimated by using the Chi-squared test (Table 2). Multivariate linear regression analysis was conducted to examine the association of reported masturbation frequency and sociodemographic factors (age, education, relationship status, religiosity), health factors (self-perceived health, depression), as well as factors related to older adults’ sexual lives (sexual activity, sexual satisfaction, and attitudes toward sexuality) (Tables 3 and 4). Results are presented as unstandardized (*b*) and standardized coefficient (*β*), and multiple correlations squared (*R*^2^).

## Results

### Sample Characteristics

Table 1 presents the sociodemographic characteristics of the four European samples by gender (weighted data). The mean age in men and women across countries was about 67 years. The level of education was highest in Norway, with 56% of men and 50% of women reporting at least some college education. The respective proportions in Portugal, the country with the lowest levels of education, were 19% for men and 16% for women. The vast majority of men reported being partnered at the time the study was conducted (ranging from 79% in Belgium to 93% in Portugal). The percentages of women being partnered were generally lower than in men, ranging from 47% in Belgium to 84% in Denmark.

**Table 1:** An overview of the sociodemographic characteristics of the sample, by country and gender (weighted data)

	Men				Women			
	Norway%	Denmark%	Belgium%	Portugal%	Norway%	Denmark%	Belgium%	Portugal%
*Age*								
60–64	35.8	27.1	28.6	36.3	35.5	28.2	38.2	34.7
65–69	34.8	31.7	37.5	35.0	34.0	31.8	33.7	36.0
70–75	29.5	41.1	33.9	28.8	30.5	40.1	28.1	29.3
*n*	634	524	485	232	636	521	505	277
*Education*								
Primary	10.6	28.6	11.2	34.6	9.4	26.4	13.1	42.4
Secondary	33.3	36.3	49.3	46.9	40.6	38.8	53.2	41.8
Tertiary	56.1	35.0	39.5	18.5	50.1	34.8	33.7	15.8
*n*	634	519	485	232	634	519	498	273
*In a relationship*								
Yes	82.2	83.5	79.1	92.7	69.3	84.4	46.8	73.1
No	17.8	16.5	20.9	7.3	30.7	15.6	53.2	26.9
*n*	631	524	485	232	635	521	504	277

Table 2 shows the level of masturbation in the past month, separately for gender and across countries. Among both men and women, there was a statistically significant difference in the frequency of masturbation across the four countries. The proportion of men and women reporting masturbation activity (at least once during the past month) was highest in Norway (65% of men and 40% of women), followed by Belgium (57% of men and 36% of women), and Denmark (53% men and 31% of women). Reporting masturbation activity was least common in Portuguese men and women (41% and 27%, respectively). Across countries, women reported less masturbation than men did. In comparison with partnered adults, masturbation activity was significantly higher among those who reported not being in a current relationship.

**Table 2:** Frequency of masturbation during the past month, in partnered and non-partnered older men and women (60–75 years) from Norway, Denmark, Belgium, and Portugal (percent, weighted data)

Masturbation frequency in the past month	Norway	Denmark	Belgium	Portugal	Sign	All partnered	All not partnered	Sign
*Men*								
None	34.7	46.6	42.7	58.1	***	46.0	25.4	***
Once	16.0	12.8	16.2	10.1		15.0	12.2	
2 or 3 times	20.2	16.5	17.8	13.3		16.5	24.4	
Once a week	11.9	11.8	13.1	9.8		10.4	20.4	
2 or 3 times a week	15.6	10.7	8.1	7.5		10.3	16.5	
Once a day or more	1.7	1.6	2.1	1.3		1.8	1.1	
*n*	611	489	461	196		1475	279	
*Women*								
None	59.8	69.1	63.8	73.3	*	67.7	58.3	***
Once	18.6	13.8	15.3	13.4		16.2	15.0	
2 or 3 times	15.7	12.7	14.7	8.2		12.0	17.8	
Once a week	4.4	3.4	3.6	2.2		2.9	5.6	
2 or 3 times a week	1.5	0.7	2.3	2.6		1.1	3.0	
Once a day or more	0.0	0.2	0.4	0.3		0.1	0.4	
*n*	602	451	436	202		1190	501	

Chi-square test of differences for country affiliation and relationship status

*p < 0.05; ***p < 0.001

Table 3 presents the findings from multiple linear regression analyses among men in the four countries on reported masturbation frequency during the past month, broken down by sociodemographic characteristics, health factors, sexual behavior, satisfaction, and attitudes toward sexuality.

**Table 3:** Masturbation frequency in men during the past month, by sociodemographic factors, health factors, sexual behavior, satisfaction, and attitudes toward sexuality, separately for each country (weighted data)

	Norway (*n* = 567)			Denmark (*n* = 449)			Belgium (*n* = 413)			Portugal (*n* = 178)		
	*B*	SE	*β*	*B*	SE	*β*	*B*	SE	*β*	*B*	SE	*β*
*Sociodemographic factors*												
Age	− .06	.01	− .17***	− .06	.02	− .17***	− .06	.02	− .17***	.01	.03	.02
Education	.21	.09	.09*	.29	.08	.16***	− .07	.11	− .03	− .35	.15	− .18*
Religiosity	.03	.04	.04	− .02	.04	− .02	− .05	.03	− .07	.01	.05	.01
Relationship status	− .26	.17	− .06	− .19	.20	− .04	− .09	.21	− .02	− .12	.42	− .02
*Health factors*												
Self-perceived health	− .08	.07	− .05	− .14	.07	− .09*	− .35	.09	− .21***	− .34	.13	− .22**
Depression	.36	.13	.12**	.28	.15	.09	.43	.13	.19***	− .11	.26	− .04
*Sexual behavior and satisfaction*												
Satisfaction with sexual activity	− .27	.08	− .19***	− .30	.06	− .23***	− .16	.07	− .13*	− .32	.14	− .22*
Intercourse activity past month	.00	.06	.00	− .16	.06	− .14**	.02	.06	.02	.25	.08	.25**
*Attitudes towards sexuality*												
Sex is for love	− .34	.07	− .20***	− .20	.06	− .14**	− .18	.06	− .14**	− .19	.10	− .15
Sexualized society	− .22	.09	− .11*	− .13	.09	− .07	− .09	.08	− .05	.08	.13	.05
Sexuality decreases with age	− .13	.07	− .08	− .21	.08	− .12**	.08	.07	.06	− .00	.09	− .00
Sexuality is beneficial	.09	.08	.04	.41	.09	.21***	.24	.09	.13**	.06	.15	.03
*R*^2^	.23			.28			.18			.13		

*p < 0.05; **p < 0.01; ***p < 0.001

Age was significantly and negativity related to masturbation frequency in Norwegian, Danish, and Belgian men (all *b*s = − 0.06). For Portuguese men, there was no relationship between age and masturbation activity. Education was significantly associated with reported masturbation, with higher levels of education associated with more masturbation in Danish (*b* = 0.29) and Norwegian men (*b* = 0.21). In contrast, among Portuguese men, higher education was related to less masturbation frequency (*b* = − 0.35). For all men, except in Norway, poorer physical health was significantly associated with less masturbation (*b*_Belgium_ = − 0.35; *b*_Portugal_ = − 0.34; *b*_Denmark_ = − 0.14). Higher levels of depression significantly predicted more frequent masturbation in Belgian (*b* = 0.43) and Norwegian men (*b* = 0.36).

Regarding sexual satisfaction, we found that greater satisfaction with one’s level of sexual activity significantly predicted lower levels of masturbation in men across all four countries (*b*_Portugal_ = − 0.32; *b*_Denmark_ = − 0.30; *b*_Norway_ = − 0.27; *b*_Belgium_ = − 0.16). Compared to Danish men, where intercourse activity was associated with less masturbation (*b* = − 0.16), the opposite relationship was found among men in Portugal (*b* = 0.25). With respect to sexual attitudes, several patterns of findings emerged. Believing that sex should happen within a loving relationship was significantly and negatively related to the level of reported masturbation for all men (*b*_Norway_ = − 0.34; *b*_Denmark_ = − 0.20; *b*_Belgium_ = − 0.18), except those in Portugal. Less permissive attitudes toward sexuality in modern society were negatively associated with masturbation activity in Norwegian men (*b* = − 0.22). Danish men who had less positive attitudes toward later life sexuality were less likely to report frequent masturbation than those who did not endorse these attitudes (*b* = − 0.21). Finally, believing that sexual activity is beneficial for older people predicted higher levels of reported masturbation in Danish (*b* = 0.41) and Belgian men (*b* = 0.24).

Results from the multiple linear regression analysis on masturbation activity for women are presented in Table 4. For women, with two exceptions, there was no relationship between masturbation and any sociodemographic or health factors. First, older age significantly predicted masturbation frequency in Danish women (*b* = − 0.03). Second, relationship status was significantly related to masturbation activity; being in a relationship predicted less masturbation frequency in Portuguese (*b* = − 0.39) and Norwegian (*b* = − 0.36) women. In Belgium and Norwegian women, greater intercourse activity predicted more masturbation (*b*_Belgium_ = 0.21; *b*_Norway_ = 0.10). Similar to men, being more satisfied with one’s level of sexual activity was negatively related to masturbation for Danish (*b* = − 0.20), Belgian (*b* = − 0.18), and Norwegian (*b* = − 0.11) women. Women in Norway and Portugal who believed that sex should only happen within loving relationships were less likely to report frequent masturbation than women with more permissive sexual attitudes (*b*_Norway_ = − 0.15; *b*_Portugal_ = − 0.12). Finally, believing that sexual activity is beneficial for older people predicted higher levels of reported masturbation in Danish (*b* = 0.16) and Belgian women (*b* = 0.13).

**Table 4:** Masturbation frequency in women during the past month, by sociodemographic factors, health factors, sexual behavior, satisfaction, and attitude towards sexuality, separately for each country (weighted data)

	Norway (*n* = 536)			Denmark (*n* = 399)			Belgium (*n* = 361)			Portugal (*n* = 179)		
	*B*	SE	*β*	*B*	SE	*β*	*B*	SE	*β*	*B*	SE	*β*
*Sociodemographic factors*												
Age	− .02	.01	− .08	− .03	.01	− .13**	− .01	.01	− .02	− .01	.02	− .03
Education	.07	.07	.04	.03	.06	.03	.08	.09	.05	.00	.11	.00
Religiosity	− .02	.03	− .03	− .00	.03	− .00	− .02	.03	− .04	.00	.03	.01
Relationship status	− .36	.11	− .16***	− .22	.13	− .09	− .39	.12	− .19***	− .27	.19	− .12
*Health factors*												
Self-perceived health	.01	.05	.01	.05	.05	.05	− .01	.07	− .01	− .04	.10	− .03
Depression	.15	.09	.08	− .05	.11	− .02	− .02	.09	− .01	− .12	.13	− .08
*Sexual behavior and satisfaction*												
Satisfaction with sexual activity	− .11	.05	− .12*	− .20	.05	− .25***	− .18	.05	− .19***	.05	.10	.05
Intercourse activity past month	.10	.04	.14*	.02	.04	.03	.21	.05	.26***	.09	.07	.14
*Attitudes toward sexuality*												
Sex is for love	− .15	.05	− .13**	− .07	.05	− .08	− .05	.05	− .06	− .12	.06	− .16*
Sexualized society	− .01	.07	− .01	− .06	.07	− .05	− .12	.07	− .09	− .05	.09	− .04
Sexuality decreases with age	− .04	.05	− .04	− .06	.05	− .07	− .08	.05	− .08	− .04	.07	− .05
Sexuality is beneficial	.10	.06	.07	.16	.06	.14**	.13	.06	.12*	.07	.09	.06
*R*^2^	.10			.14			.15			.09		

*p < 0.05; **p < 0.01; ***p < 0.001

## Discussion

In this European four-country study, we assessed several sociodemographic, health, attitudinal, and sexual behavioral factors associated with reported masturbation frequency among men and women aged 60–75 years. Despite marked cross-cultural and gender differences in masturbation frequency, predictors of masturbation were in most instances more similar than different across the four countries. Satisfaction with the level of sexual activity was a significant negative predictor of masturbation in Norwegian, Danish, and Belgian men and women, and Portuguese men. Another important predictor of frequent masturbation was sexual attitudes. Specifically, attitudes reflecting the idea that sex is legitimized by love were associated with masturbation in Norwegian, Danish, and Belgian men, and Norwegian and Portuguese women. While age, education, self-perceived health, and depression were significantly related to men’s reported masturbation, few sociodemographic and health factors were associated with masturbation activity among women.

Satisfaction with sexual activity was significantly related to masturbation in almost all countries. Men and women in Norway, Denmark, and Belgium, and men in Portugal were less likely to report frequent masturbation if they were satisfied with their level of sexual activity. The central role of sexual contentment in predicting solitary sexual activity may reflect older adults’ tendency to view masturbation as a second-best alternative that is only needed if one desires more sex or partnered sex is not satisfying. Although this finding supports the idea that masturbation functions as a substitute among contemporary older populations, it is possible that this will not be the case for the coming generations. Findings from Finland and Germany indicate cultural changes in the meaning of masturbation, with younger generations increasingly considering it as an independent source of experiencing sexual pleasure (Dekker & Schmidt, 2003; Kontula & Haavio-Mannila, 2003).

Similar to previous findings, the association between intercourse and masturbation frequency was not unequivocal (Regnerus et al., 2017; Rowland et al., 2020). As with Gerressu et al. (2008), we found a gender-specific pattern where, among Norwegian and Belgian women, more frequent intercourse increased the likelihood of frequent masturbation (reflecting the complementary model), while the opposite relationship was found among Danish men (in line with the compensatory model). In contrast to this pattern was the finding in Portuguese men, where more intercourse activity was related to higher levels of masturbation. Although this finding may represent a difference in sexual culture in southern Europe, it is more likely that it reflects a sample selection bias, given a less reliable sampling method, a high refusal rate, and a much lower response rate for the Portuguese sample than for the samples in Norway, Denmark, and Belgium.

Another key finding points to the important role of sexual attitudes in predicting sexual behavior in aging men and women. Sexual attitudes mirror prevailing sociocultural norms and the attached meaning of sexual behavior within a cultural context (Masters et al., 2013). We investigated several sexual attitudes as possible predictors of masturbation and found that attitudes reflecting the idea that sex is legitimized by love were negatively associated with masturbation in Norwegian, Danish, and Belgian men, and in Norwegian and Portuguese women. This finding may reflect the prevailing heterosexual sexual script, where sexual behavior is legitimized by romantic love (also termed the “love ideology”) (Francoeur & Noonan, 2004; Gagnon & Simon, 2005; Træen & Lewin, 2008). According to this script, “good” sexuality is contextualized within intimate relationships, where partnered sex symbolizes mutual love and commitment (Fileborn et al., 2017; Gagnon & Simon, 2005; Hinchliff & Gott, 2004; Træen & Lewin, 2008). Within this love script, there exists little space for sexual self-pleasuring (Hogarth & Ingham, 2009). Disapproval of sex without love among older adults seems to reflect this traditional script and the idea that partnered sex is superior to masturbation. Hence, masturbation signifies something suboptimal and unnecessary, especially if one has access to the “real deal” (Træen et al., 2019). Moreover, practicing sexual self-pleasuring in a relationship might be associated with the fear that the partner may misconstrue the behavior as a sign of personal undesirability and sexual incompetence (Onar et al., 2020). Attitudes reflecting the notion that sexuality decreases with older age and that society has become too sexualized were also negatively related to masturbation frequency, but only in men from Norway and Denmark. In contrast to these less permissive attitudes, Danish and Belgian men and women who believed that sexual activity is beneficial for older people were more likely to report frequent masturbation than those who did not endorse these attitudes. This finding is consistent with previous research showing a positive link between more liberal attitudes/values and reported masturbation (Das et al., 2009; Gerressu et al., 2008).

Overall, more sociodemographic factors were predictive of men’s masturbation than women’s. As found by others (Corona et al., 2010; Lee et al., 2016; Lindau et al., 2007; Mercer et al., 2013; Palacios-Ceña et al., 2012; Richters et al., 2014; Schick et al., 2010), older age was negatively associated with masturbation in Norwegian, Danish, and Belgian men, and Danish women. However, as with previous cross-sectional data, it was not possible to assess whether this reflected an age or cohort effect. Regarding the level of education, we found some cultural-specific patterns. While higher levels of education increased the likelihood of masturbation frequency in northern European men (Norway and Denmark), southern European men (Portugal) with higher levels of education were less likely to report frequent masturbation. Being socialized in an environment influenced by traditional and religious structures repressing sex education (Francoeur & Noona, 2004), older educated Portuguese men may have internalized more normative constraints inhibiting sexual self-pleasure. In contrast, among Norwegian and Danish men who were socialized in the sex-liberal Nordic countries, with open discourses on sexual issues and broad dissemination of sex education, pornography, and sex literature (Francoeur & Noona, 2004), higher education may have shaped masturbation habits by diminishing health-related fears and guilt about masturbation (Kontula & Haavio-Mannila, 2003).

In terms of health, we observed a negative association between self-estimated health and reported masturbation among older men in Denmark, Belgium, and Portugal. Previous evidence on the relationship between general health and masturbation has been mixed (Das, 2007; Lee et al., 2016; Lindau et al., 2007; Schick et al., 2010). One possibility for the inconsistency is that the association might be confounded by older men’s sexual difficulties and sexual desire. While for some men poor health may negatively affect their overall sexual functioning and sexual interest, hence influencing both partnered and solo sex, others with reduced sexual function but high desire may replace partnered sex by increased autoerotic behavior. Future research is needed to address this moderation hypothesis.

In addition to self-rated general health, we assessed the relationship between negative mood and solitary sexual activity. Interestingly, our findings demonstrated a significant association between depression and masturbation among Norwegian and Belgian men; the higher the level of depression, the more likely the reported masturbation. Although this finding seems counterintuitive, it is consistent with previous research (Cyranowski et al., 2004; Frohlich & Meston, 2002; Rowland et al., 2020). One assumption has been that when feeling depressed increased masturbation might reflect a self-soothing strategy, where solo sex functions as a reliable way to make oneself feel better (Frohlich & Meston, 2002). Although self-stimulation when feeling depressed may be self-soothing in the short term, it does not necessarily enhance mood as masturbation also seems to reinforce feelings of loneliness and isolation (Bancroft et al., 2003).

Finally, regarding relationship status, we found that women in Norway and Portugal were less likely to report frequent masturbation if they were in a current relationship. This finding seems to corroborate results from previous studies among varied age groups (DeLamater & Moorman, 2007; Regnerus et al., 2017; Rowland et al., 2020; Schick et al., 2010). It seems probable that since partnered adults may anticipate the opportunity of having sex with their committed partner, they wish to channel their sexual desire into their sexual relationship and/or do not feel the need for masturbation (Regnerus et al., 2017).

### Strengths and Limitations

Our survey had several strengths, including the large probability-based samples and the use of similar sampling methods, identical measures, and age cohorts across four European countries. Several limitations, however, should also be acknowledged. The sample size in Portugal, as well as the response rate, was much lower than in the other countries. Due to a less reliable sampling method, an overrepresentation of urban individuals, and a high refusal rate, the selection bias was possibly most substantial among Portuguese participants (Boughner, 2010). Overall, this gives rise to questions relating to the Portuguese samples’ representativeness and its comparability with the samples from Norway, Denmark, and Belgium, which should be taken into consideration when evaluating the study findings. A second limitation pertains to the item formation. In this study, a preexisting one-item indicator was used to measure reported masturbation frequency (ELSA; Lee et al., 2016). Because the question did not specifically refer to solo masturbation, we cannot be sure about the extent to which the results reflected only solo masturbation or both partnered and solo masturbation. However, both the wording of the item (“How often did you masturbate in the past month”) and the context (following a question asking about sexual intercourse frequency) provide some reassurance that the participants interpreted it as a question about solo sexual activity. Further, satisfaction with sexual activity was measured by the question: “How satisfied are you with the current level of sexual activity in your life, in a general way?” Considering the lack of defining sexual activity when asking about participants’ levels of sexual satisfaction, it is likely that participants used divergent definitions when they evaluated their levels of sexual satisfaction (Regnerus et al., 2017). Some might limit sexual activity to solely partnered sex, while others might incorporate solo sexual activities. Yet, a study that investigated the concept of sexual satisfaction among German women found that most of the variance in satisfaction with sex life in general was explained by sexual satisfaction through intercourse and intercourse frequency (Philippsohn & Hartmann, 2009). Another important limitation was that the survey did not assess the role of pornography use. Although our samples might have been biased toward individuals with more liberal and open views about sexuality (Boughner, 2010; Dunne et al., 1997; Strassberg & Lowe, 1995), as sexual self-stimulation is a stigmatized and sensitive topic that might be embarrassing to older individuals, the prevalence of masturbation may still have been underestimated due to social desirability. How possible volunteer bias and social desirability influenced the associations is uncertain, but it may restrict the generalizability of our findings (Boughner, 2010). Finally, due to the cross-sectional design, conclusions about possible causal relationships are not warranted.

### Conclusion

Previous research on solitary sexual activity highlighted the links between cultural-dependent sexual socialization processes and masturbation and how these change across different generations (Kontula & Haavio-Mannila, 2003). The present findings seem to support the validity of the compensatory model in contemporary older adults (Štulhofer et al., 2018); aging women and men appear to be less likely to report masturbating if they are content with their level of sexual activity and if they disapprove of sex without love. Our findings highlight the importance of positive sexual attitudes and education for enhancing sexual self-gratification in later life. As masturbation is a free, safe, and independent way to experience sexual pleasure, it has substantial potential for sexual health promotion in the aging population (Kontula & Haavio-Mannila, 2003).
